# Different Reactions to Adverse Neighborhoods in Games of Cooperation

**DOI:** 10.1371/journal.pone.0035183

**Published:** 2012-04-23

**Authors:** Chunyan Zhang, Jianlei Zhang, Franz J. Weissing, Matjaž Perc, Guangming Xie, Long Wang

**Affiliations:** 1 State Key Laboratory for Turbulence and Complex Systems, College of Engineering, Peking University, Beijing, China; 2 Theoretical Biology Group, University of Groningen, Groningen, The Netherlands; 3 Department of Physics, Faculty of Natural Sciences and Mathematics, University of Maribor, Maribor, Slovenia; Universidad Carlos III de Madrid, Spain

## Abstract

In social dilemmas, cooperation among randomly interacting individuals is often difficult to achieve. The situation changes if interactions take place in a network where the network structure jointly evolves with the behavioral strategies of the interacting individuals. In particular, cooperation can be stabilized if individuals tend to cut interaction links when facing adverse neighborhoods. Here we consider two different types of reaction to adverse neighborhoods, and all possible mixtures between these reactions. When faced with a gloomy outlook, players can either choose to cut and rewire some of their links to other individuals, or they can migrate to another location and establish new links in the new local neighborhood. We find that in general local rewiring is more favorable for the evolution of cooperation than emigration from adverse neighborhoods. Rewiring helps to maintain the diversity in the degree distribution of players and favors the spontaneous emergence of cooperative clusters. Both properties are known to favor the evolution of cooperation on networks. Interestingly, a mixture of migration and rewiring is even more favorable for the evolution of cooperation than rewiring on its own. While most models only consider a single type of reaction to adverse neighborhoods, the coexistence of several such reactions may actually be an optimal setting for the evolution of cooperation.

## Introduction

Cooperation is a fascinating area of research since it touches upon so many different disciplines, ranging from biology to economics, sociology, and even theology [1]. The fact that in human societies cooperative behavior is common among unrelated people is puzzling from evolutionary point of view, since cooperation can easily be exploited by selfish strategies. Evolutionary game theory [2]–[4] provides a theoretical framework to address the subtleties of cooperation among selfish individuals. In particular, the prisoner's dilemma [5], [6] is a paradigm example for studying the emergence of cooperation in spite of the fact that self-interest seems to dictate defective behavior.

Past research has identified several key mechanisms (comprehensively reviewed in [7]) that promote the evolution of cooperation. In particular, spatial reciprocity [8] has launched a spree of activity aimed at disentangling the role of the spatial structure by the evolution of cooperation. The seminal works in this area focused on regular graphs and lattices [8]–[18]. Later attention shifted to more complex networks [19], [20], and, in particular, to scale-free networks. Evolutionary games on graphs and networks are thoroughly reviewed in [21]. More recent studies have elaborated on various aspects, including the dynamical organization [22], clustering [23] and mixing patterns [24], [25], as well as memory [26], robustness [27], phase transitions [28] and payoff normalization [29], [30].

While considering population structure is an important step for understanding the evolution of cooperation, a crucial ingredient is still missing. In real social networks the interaction structure is frequently not static but evolving in concern with the behavior of the interacting agents. As reviewed in [31] a vibrant new research area is emerging that studies the joint evolution of interaction structure and behavior. Many models have focused on the way players make (or break) links in reaction to the degree of cooperation they experienced from their interaction partners [32]–[46]. Other models have considered the possibility to leave uncooperative neighborhoods [47]–[54]. It is plausible that both mechanisms can promote the evolution of cooperation, but it is not obvious which of the two mechanisms is more efficient. Moreover, it is not self-evident that all individuals use the same rules for changing their interaction network in response to adverse conditions. In fact, everyday experience tells us that different people may react quite differently when they find themselves in a bad neighborhood: while some tend to migrate to another location, others tend to stay put and instead search for new friends (or get rid of old friends) in order to improve the situation.

Motivated by such considerations, we study the joint evolution of cooperation and interaction structure in the prisoner's dilemma game and in the snowdrift game. The players are of two types that differ in the way they react to an adverse neighborhood. A fixed fraction 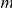 of the players consists of ‘migrants’, who in proportion to the number of defectors in their neighborhood tend to migrate to another (unoccupied) position in the network. The complementary fraction 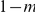 of the players consists of ‘rewirers’, who have the tendency to break their links with defectors and subsequently to reattach the free links to other players. By changing the parameter 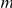, our model allows to transverse smoothly from an adaptive linking model (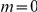) to a migratory model (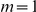). In between these two extremes, we have a situation where different players react differently when finding themselves in an adverse neighborhood.

## Results

Our analysis is based on the prisoner's dilemma game and the snowdrift game, two paradigm models for the evolution of cooperation. We label the payoff parameters in line with the conventions for the prisoner's dilemma [5]: a cooperating player receives the “reward” 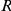 in case of mutual cooperation and the “sucker's payoff” 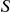 in case of being defected; a defecting player receives the “temptation to defect” 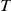 when the other player cooperates and the “punishment” 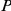 in case of mutual defection. By definition, a prisoner's dilemma game satisfies the payoff relationships 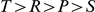. When played as a one-shot game in a well-mixed population, defect is the only evolutionarily stable strategy; despite of the fact that the payoff 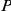 to both players can be considerably smaller than the payoff 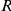 for mutual cooperation. The snowdrift game is characterized by 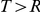 and 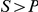. When played as a one-shot game in a well-mixed population, none of the two pure strategies is evolutionarily stable and a mixed strategy is expected to result [16]. Without loss of generality, we normalize 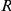 and 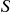 to 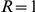 and 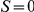. In all our graphs, 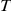 is systematically varied from 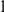 to 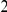. Hence, the game considered is a prisoner's dilemma game if 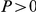 and a snowdrift game if 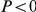.

**Figure 1:**
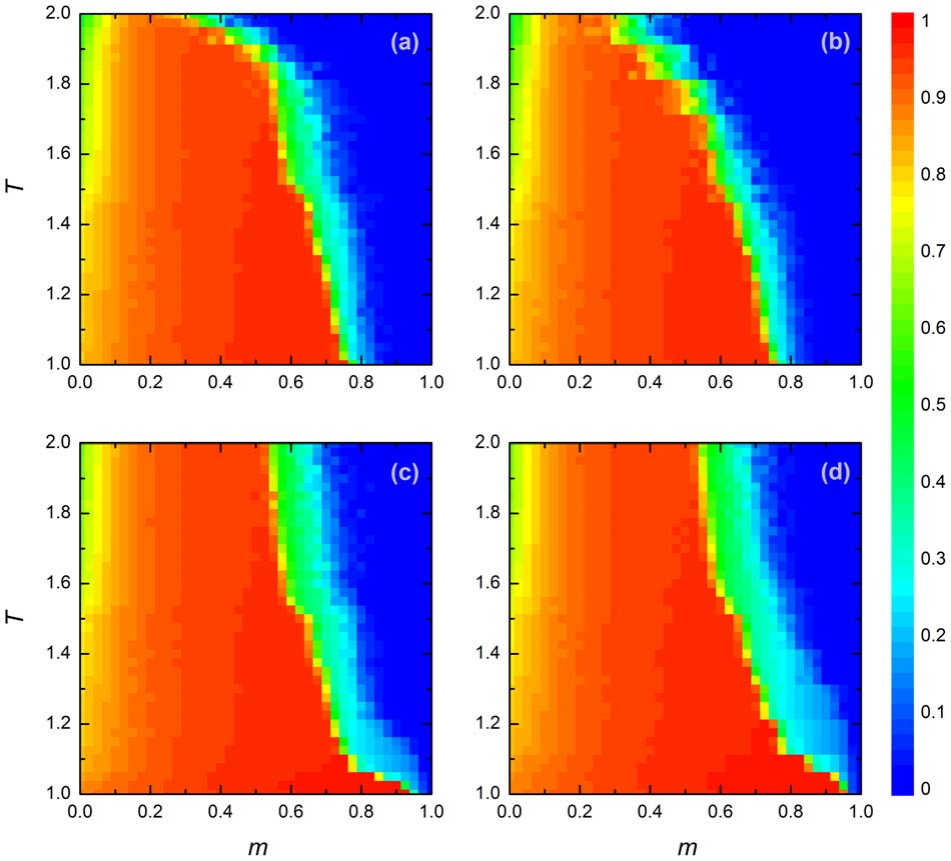
Stationary density of cooperators in dependence on the fraction of players reacting to adverse conditions by migration (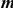) and the payoff parameter (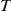). Panels (a) and (b) depict the outcome for the prisoner's dilemma game with 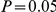 and 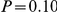, respectively, while panels (c) and (d) depict the outcome for the snowdrift game with 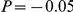 and 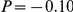, respectively. Other payoff parameters are 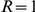 and 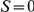 in all four panels. The density of cooperators in the stationary state (after 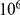 iteration steps) is color-coded from blue (full defection) to red (full cooperation), as indicated on the right of the figure. If all players are “migrants” (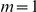; right-hand border of each panel), defection is clearly dominant, while intermediate levels of cooperation evolve if all players are “rewirers” (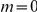; left-hand border of each panel). In all types of game, the highest level of cooperation evolves in mixed populations consisting of both migrants and rewirers. In this figure, the density of occupied nodes is 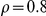.

Fig. 1 shows how for four values of the payoff parameter 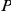 the level of cooperation evolves in relation to the temptation 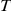 to defect and the relative frequency 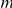 of players reacting to adverse conditions by migration. By and large, the outcome is very similar in all four cases. Cooperation is more difficult to achieve for larger values of 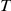, but in general the outcome is dominated by the parameter 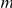. If migration is the only reaction to adverse conditions (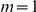; right-hand border of each panel), cooperation goes extinct not only in the prisoner's dilemma games (upper panels in Fig. 1) but also in the snowdrift games (bottom panels in Fig. 1). In contrast, cooperation can reach high levels or even go to fixation if all players react to adverse conditions by rewiring (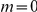; left-hand border of each panel). Interestingly, a combination of migration and rewiring is most favorable for the evolution of cooperation. For a broad range of 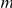-values (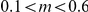), cooperation tends to fixation, even in case of a relatively large temptation 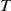 to defect. Apparently, cooperation is favored if a certain fraction of the players choose for a complete reset of their interactions when surrounded by defectors, while too high levels of migration mix up the population to such an extent that local structures providing a foothold for cooperation cannot develop.

To further analyze the mechanisms enhancing or impeding cooperation, we performed extensive numerical simulations for the case 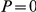. This is a border case that is sometimes called a “weak” prisoner's dilemma game [6]. In general, the weak form of the prisoner's dilemma game can have other properties than the strong form [55], but Fig. 1 clearly demonstrates that this is not the case in our model.

**Figure 2:**
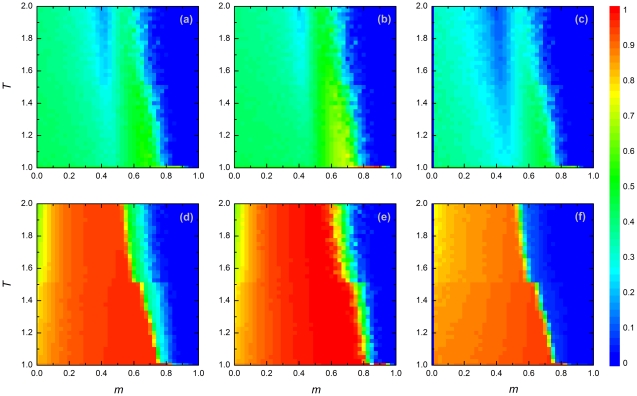
Stationary density of cooperators amongst different type of players. Panels depict results for all players (a,d), for players adopting rewiring (b,e), and for players adopting migration (c,f), each for two different population densities [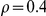 in (a,b,c) and 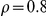 in (d,e,f)]. As in Fig. 1, each panel shows the outcome in dependence on the fraction of players reacting to adverse conditions by migration (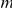) and the payoff parameter (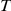), color coded as indicated on the right of the figure. Other payoff parameters are 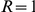 and 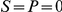 in all six panels. See main text for further details.

Fig. 2 shows how the evolution of cooperation is affected by population density. At low densities (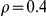; upper panels), cooperation does not get off the ground and at best stays at the initial level. If the majority of the population reacts to adverse conditions by migration, cooperation goes extinct. Similar results were obtained when the value of 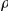 was smaller than about 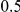. If the population density is too small, players only have few interaction partners, making it difficult for cooperators to form local clusters enforcing their success.

Up to now, we have focused on the behavior of the system as a whole. We will now zoom in a bit and study the different types of player in more detail. The middle and right panels of Fig. 2 demonstrate that in the stationary state the strategy choice (cooperate versus defect) of a player becomes associated with the player's reaction to adverse conditions. In fact, players adopting rewiring (Fig. 2(b)(e)) show a markedly higher tendency to cooperate than players adopting migration (Fig. 2(c)(f)). The difference in cooperation tendency between both types of player is smallest at low population densities (here 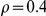), where for neither type of player the relative frequency of cooperation exceeds 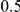. One reason for this may be that the migration of players provides an opportunity for defectors to invade and destroy the sparse cooperative clusters in the scattered population, essentially creating a situation comparable to well-mixed conditions. In the low-density scenario, cooperation is only sustained (at intermediate level) when the exploitation from defectors is not too strong. At high densities, the situation is markedly different. Even for relatively large values of 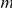, there is a boost of cooperation even for large values of 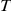. Still, players adopting migration do worse than those adopting rewiring.

**Figure 3:**
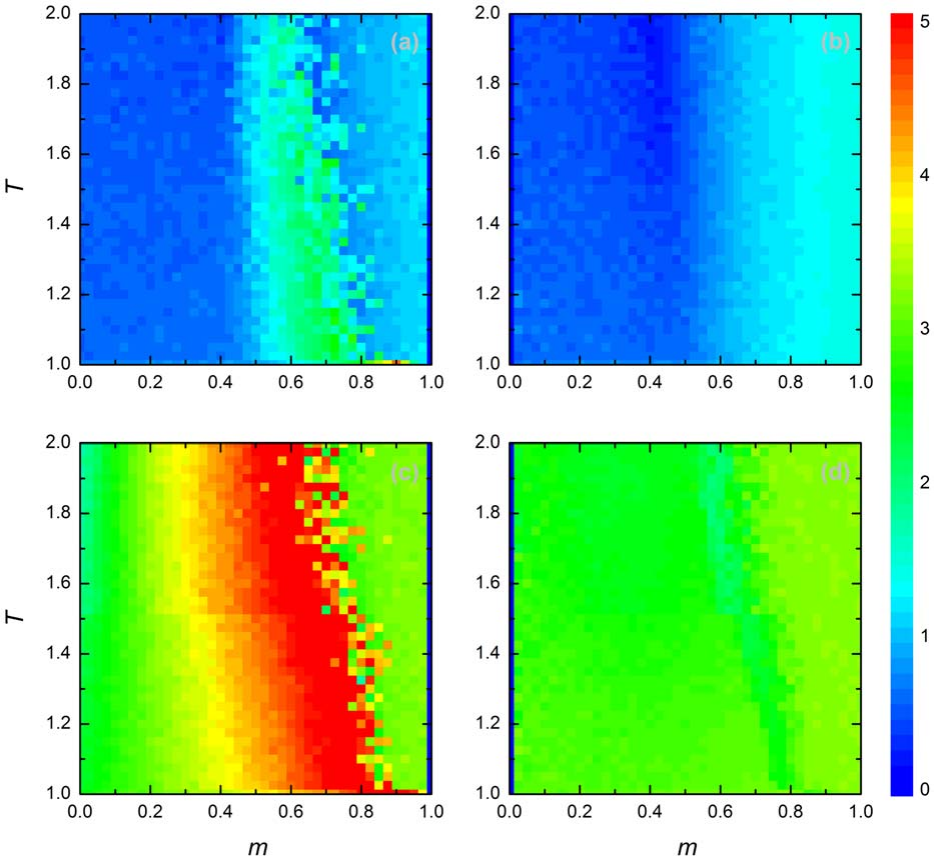
Average degree of different types of players. Panels depict results for players adopting rewiring (a,c) and for players adopting migration (b,d), each for two different population densities [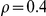 in (a,b) and 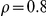 in (c,d)]. The average initial degree of each occupied node (player) was 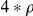. Graphical conventions and payoff parameters are the same in Fig. 2, only that the color bar encodes the average degree in the stationary state. See main text for further details.

Fig. 3 shows how the reaction of a player to adverse conditions affects the player's degree of connectedness in the evolved stationary population. It is obvious that the topology of a player's neighborhood is at least partly shaped by the player's behavioral choices. It has been shown that the adaptive interplay between the players' strategies and the underlying network can lead to the emergence of heterogeneity from an initially homogeneous connectivity structure [8]. As before, the difference in connectivity between players adopting migration and players adopting rewiring is quite small at low population density (upper panels in Fig. 3). This difference becomes much more pronounced at high density. For players adopting rewiring (left panels in Fig. 3), the average degree first increases with 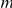 (to reach highest levels for 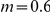), subsequently decreasing with a further increase of 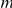. In contrast, the average degree of players adopting migration only marginally depends on 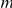, staying close to the initial value of 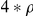. For most parameter combinations, players adopting rewiring have a higher degree than players adopting migration. Presumably, rewiring leads to an accumulation of links between cooperators and thereby the formation of tightly connected cooperative clusters.

**Figure 4:**
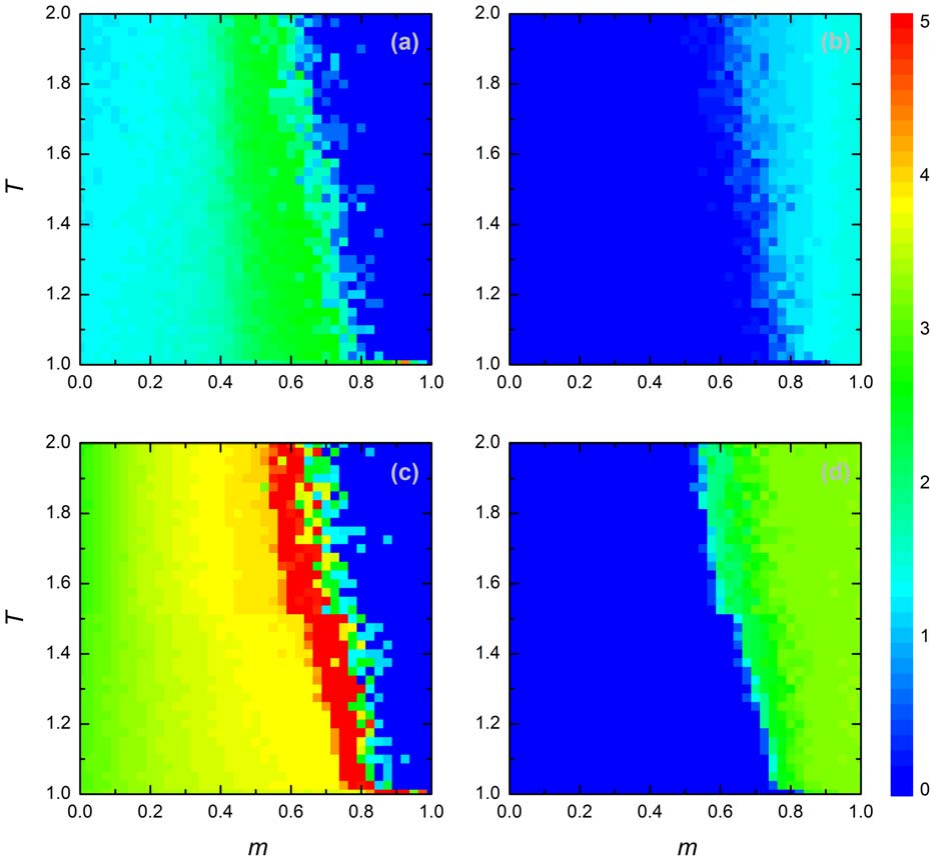
Average degree of players depending on their strategies. Panels depict results for cooperators (a,c) and defectors (b,d), each for two different population densities [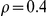 in (a,b) and 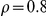 in (c,d)]. Graphical conventions and payoff parameters are the same in Fig. 3. See main text for further details.

This interpretation is corroborated by Fig. 4, which shows the difference in connectedness between cooperating and defecting individuals. The regions in parameter space where a high level of cooperation evolved (Fig. 2(d)) corresponds to those regions where cooperators are tightly connected (i.e. where cooperators have a high degree). As before, connectedness is low in the low-density situation (where cooperation did not get off the ground) and much higher (at least for cooperators) in the high-density situation. For low and intermediate values of 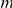, the connectedness of cooperators is markedly higher than the connectedness of defectors. The opposite is the case a high values of 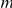.

**Figure 5:**
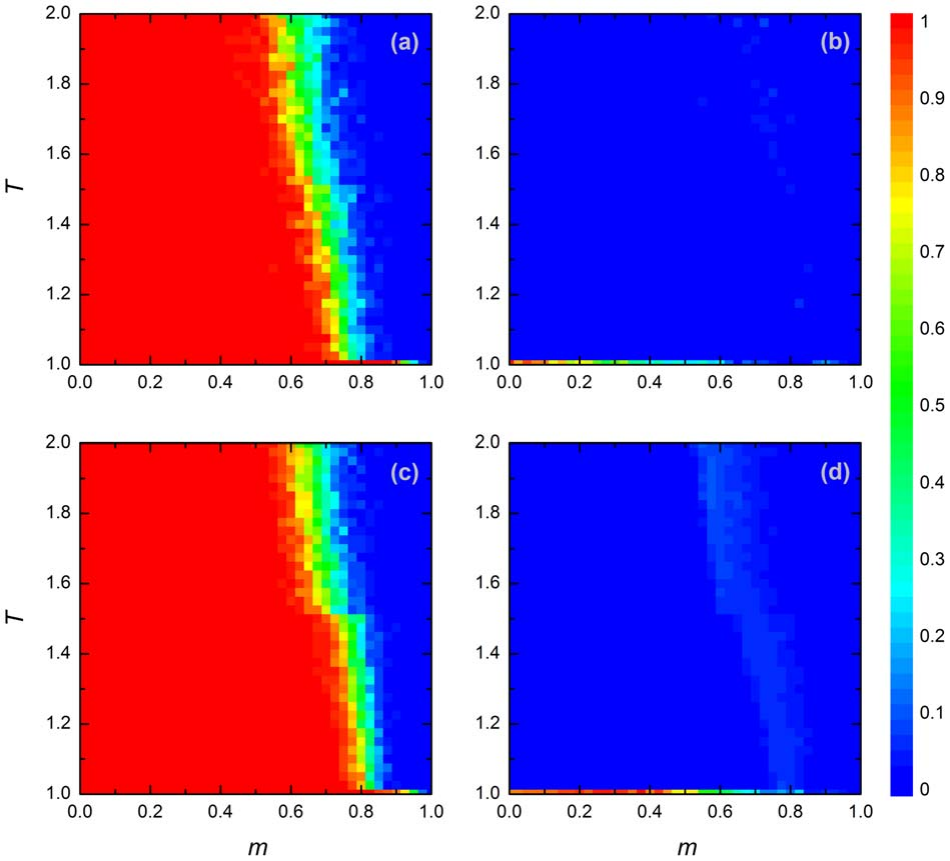
Stationary density of cooperators in different neighborhoods. Panels depict results for the neighborhood of cooperators (a,c) and the neighborhood of defectors (b,d), each for two different population densities [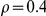 in (a,b) and 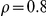 in (c,d)]. Graphical conventions and payoff parameters are the same as in Fig. 2. See main text for further details.

Finally, we consider the differences in the degree of cooperation experienced by cooperators and defectors, respectively (Fig. 5). Irrespective of population density and other parameters, defectors always ended up in adverse neighborhoods. In contrast, cooperators tended to interact only with other cooperators - at least as long as the fraction of players adopting rewiring was not too small (i.e. for 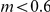). For large values of 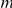, cooperative clusters did not emerge, corresponding to the collapse of cooperation under these conditions.

Overall, our results confirm the importance of the formation of cooperative clusters. If population densities are not too low and if sufficiently many individuals adopt rewiring, cooperative clusters can emerge even under unfavorable conditions (e.g. a large value of 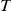). Once clusters of cooperators have formed, selection against defective partners can effectively shield clusters of cooperators from the invasion of defectors. This is so because cooperators within the cluster attract interactions with cooperators at the cluster boundary. In this way the payoffs of cooperators both inside the cluster and at its fringe are enhanced and interactions with defectors are avoided. Defectors surrounding clusters of cooperators have limited opportunities for exploitation, allowing clusters of cooperators to expand. Thus, clusters uphold cooperative behavior even if the temptation to defect is large. Conversely, defectors are unable to claim lasting benefits from occupying the clusters, simply because they become very weak as soon as all the neighbors of the defecting cluster become defectors themselves.

## Discussion

In line with other studies, we have shown that in a network environment cooperation in social dilemmas does readily evolve if players have the opportunity to change their local interaction structure when surrounded by non-cooperative neighbors. Among the two reactions to adverse conditions considered, rewiring was clearly more favorable for the evolution of cooperation than migration. This was even the case in a model where the costs of rewiring and emigration were the same (zero), while migration will often be more costly in natural settings. Interestingly, the highest degree of cooperation evolved when the player population was polymorphic in the sense that both types of reaction to adverse neighborhoods (rewiring and migration) were present in non-negligible frequencies. We interpret this finding by the interplay of two factors: while migration induces a certain mixing of the population due to increased interaction ranges of the migrating players, rewiring may lead to strongly heterogeneous interactions networks, which are tightly associated with flushing cooperative states [38].

In our model, the reaction to adverse conditions was assumed as a fixed property of each player. Hence, this reaction did not evolve. Our results suggest that the joint evolution of the strategies in the cooperation game and the reaction to adverse condition would lead to a polymorphism in the reaction to adverse neighborhoods. It remains to be seen whether this is indeed the case.

## Methods

All simulations were run on a lattice of 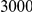 nodes with periodic boundary. In each simulation, a fraction 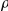 of the lattice nodes was occupied by 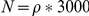 players, who initially were distributed randomly over the lattice. Initially, each player was connected with all players on adjacent lattice nodes. Accordingly, the initial degree of each player ranged from 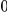 to 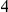, with an average of 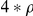.

Throughout a simulation, each player had the fixed status of either adopting migration or rewiring when confronted with adverse conditions. This status was assigned to players at random at the start of a simulation, with 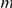 being the fraction of migrants. In addition, players could at each time be classified as either cooperators or defectors in the cooperation game, but the strategy of each player could change during a simulation due to payoff-based learning. Initially, the strategies 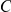 and 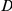 were randomly assigned to the players with equal probability.

To simulate evolution, we employed event-based asynchronous updating where interactions, rewiring and migration all occurred on the same time scale. Whenever an “event” occurred, a focal player 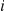 was chosen at random. This player had pairwise interactions with all “neighbors” (that is, all players connected with 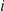), yielding a sum 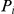 of all payoffs. For a randomly selected neighbor 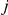 of 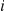, the payoff 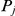 was determined in a similar way. Based on the payoff difference 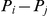, the focal player switches to j's strategy with probability 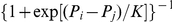. If 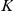 is large, payoff differences do not matter much for the direction of strategy change, while such differences are decisive in case of a small value of 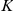. Throughout this work we set 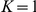, indicating that strategies of better performing players are readily, though not always, adopted.

Following the game interactions and the strategy change phase, the focal player 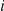 reconsiders its interaction structure. If the focal individual adopts rewiring, it will cut a random tie with a defecting neighbor with probability proportional to the number of defectors; subsequently the free link will be reattached to another player randomly chosen from the entire population. If the focal player adopts migration, it will migrate to a randomly chosen empty target site with probability proportional to the number of defectors in its neighborhood; at the new site, it will establish new links with all players occupying adjacent sites on the network.

During a full iteration, the above process was repeated 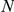 times. Hence on average each player was in the focal role once per iteration, and each player had on average once the opportunity to pass its strategy to one of its neighbors. The process was repeated until a stationary state was reached, where the distribution of strategies and the characteristics of neighborhoods did not change any more. Typically we ran each simulation for 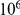 steps. For each parameter combination we ran 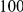 replicate simulations. The results reported are averages over these replicates.
